# Association between obesity and mortality in critically ill COVID-19 patients requiring invasive mechanical ventilation: a multicenter retrospective observational study

**DOI:** 10.1038/s41598-023-39157-8

**Published:** 2023-07-24

**Authors:** Keiichiro Shimoyama, Akira Endo, Takashi Shimazui, Takashi Tagami, Kazuma Yamakawa, Mineji Hayakawa, Takayuki Ogura, Atsushi Hirayama, Hideo Yasunaga, Jun Oda

**Affiliations:** 1 Department of Emergency and Critical Care Medicine, Tokyo Medical University Hospital, 6-7-1, Nisi-Shinjuku, Shinjuku-ku, Tokyo, 160-0023 Japan; 2 Trauma and Acute Critical Care Center, Tokyo Medical and Dental University Hospital, 1-5-45 Yushima, Bunkyo-ku, Tokyo, 113-8519 Japan; 3 Department of Acute Critical Care Medicine, Tsuchiura Kyodo General Hospital, 4-1-1, Otsuno, Tsuchiura, Ibaraki 300-0028 Japan; 4 Department of Emergency and Critical Care Medicine, Kimitsu Chuo Hospital, 1010 Sakurai, Kisarazu, Chiba 292-8535 Japan; 5 Department of Emergency and Critical Care Medicine, Nippon Medical School Musashikosugi Hospital, 1-383 Kosugimachi, Nakahara-ku, Kawasaki, Kanagawa 211-8533 Japan; 6 Department of Emergency Medicine, Osaka Medical and Pharmaceutical University, 2-7 Daigakumachi, Takatsuki, Osaka 569-8686 Japan; 7 Department of Emergency Medicine, Hokkaido University Hospital, N14W5, Kita-ku, Sapporo, 060-8648 Japan; 8 Department of Emergency Medicine and Critical Care Medicine, Tochigi Prefectural Emergency and Critical Care Center, Imperial Gift Foundation SAISEIKAI, Utsunomiya Hospital, 911-1 Takebayashi-machi, Utsunomiya, Tochigi 321-0974 Japan; 9 Public Health, Department of Social Medicine, Graduate School of Medicine, Osaka University, 2-15, Yamadaoka, Suita, Osaka 565-0871 Japan; 10 Department of Clinical Epidemiology and Health Economics, School of Public Health, The University of Tokyo, 7-3-1 Hong, Bunkyo-ku, Tokyo, 113-8655 Japan; 11 Department of Traumatology and Acute Critical Medicine, Graduate School of Medicine, Osaka University, 2-15, Yamadaoka, Suita, Osaka 565-0871 Japan

**Keywords:** Diseases, Infectious diseases, Viral infection

## Abstract

This study aimed to determine whether obesity and disease outcomes are associated in patients with critically-ill coronavirus disease 2019 (COVID-19) requiring invasive mechanical ventilation (IMV). This retrospective observational study using Japanese multicenter registry data included COVID-19 patients who required IMV and were discharged between January and September 2020. The patients were divided into the obese (body mass index [BMI] ≥ 25 kg/m^2^) and nonobese (BMI < 25 kg/m^2^) groups. Logistic regression models were used to analyze the association between obesity and disease outcomes. The primary outcome was in-hospital mortality; the secondary outcome was venovenous extracorporeal membrane oxygenation (VV-ECMO) implementation. Altogether, 477 patients were enrolled (obese, n = 235, median BMI, 28.2 kg/m^2^; nonobese, n = 242, median BMI, 22.4 kg/m^2^). Obesity was significantly associated with lower in-hospital mortality in the unadjusted logistic regression model (odds ratio 0.63; 95% confidence interval, 0.42–0.97; p = 0.033), but not with mortality in the adjusted logistic regression model using age, sex, and Charlson Comorbidity Index as covariates (p = 0.564). Obesity was not associated with VV-ECMO implementation in both unadjusted and adjusted models (unadjusted, p = 0.074; adjusted, p = 0.695). Obesity was not associated with outcomes in COVID-19 patients requiring IMV. Obesity may not be a risk factor for poor outcomes in these patients.

## Introduction

Since 2019, the novel coronavirus disease 2019 (COVID-19) has been a recurrent global pandemic. As of January 2023, 664,873,023 people have been infected and 6,724,248 people, corresponding to 1% of all patients with the disease, have died globally^1^. Additionally, undiagnosed COVID-19 victims have been reported^2^, which has become a major global public health concern. Approximately 20.2% hospitalized COVID-19 patients required invasive mechanical ventilation (IMV) and 26.5% of them died^3^. Furthermore, in a meta-analysis, the mortality rate of these patients was reported to be as high as 56%^4^. Despite the advancements in the development of vaccines and novel therapeutic agents, treatment options that dramatically improve the condition of severe ill patients are limited. Therefore, the identification of prognostic factors in COVID-19 patients requiring IMV would help clinicians when considering treatment intensity and additional therapeutic options, and it may provide new insights into the pathogenesis of COVID-19.

As several studies have reported that obesity is considered to be a risk factor for worsening outcomes in entire COVID-19 patients, increasing the need for IMV^5^, intensive care unit (ICU) admission^6,7^, and mortality risk^8–10^. However, especially in critically ill COVID-19 patients, the association between obesity and mortality is still unclear. A previous report conducted in the United States and Israel suggested that obesity was associated with increased mortality in the cohort of intubated and nonintubated COVID-19 patients who required ICU treatment^11^. Contrarily, a small cohort study in ICU, which also included both intubated and nonintubated patients, conducted in France suggested that obesity was not associated with mortality in COVID-19 patients^12^. There is no study on obesity and the disease prognosis, including mortality, only in the intubated COVID-19 patients, who are considered the severer population. Additionally, while these investigations suggested an inconsistent association between obesity and mortality in COVID-19 patients among western populations, there has been no sufficient large-size cohort study among Asian populations.

To address this knowledge gap, we aimed to examine the association between obesity and mortality in Asian COVID-19 patients requiring IMV using data from a Japanese multicenter registry.

## Methods

### Study design and settings

This was a retrospective observational cohort study using the data of Japanese multicenter research on COVID-19 by assembling real-world data (J-RECOVER) study registry^13^. Briefly, the J-RECOVER study is a multicenter study involving 66 research and teaching hospitals in Japan, which collected clinical information on patients with a laboratory-confirmed severe acute respiratory syndrome coronavirus 2 (SARS-CoV-2) infection and were discharged between January 1 and September 31, 2020. The J-RECOVER study registry data consists of diagnosis procedure combination (DPC) data, clinical data extracted from medical records by researchers at each facility, and all laboratory data conducted during hospitalization^13^. The DPC data are tied to the comprehensive payment system for Japanese in more than 1600 acute care hospitals and are submitted monthly to the Ministry of Health, Labor, and Welfare^14^.

This manuscript was described in accordance with the Strengthening the Reporting of Observational Studies in Epidemiology Statement (STROBE). The study was approved by the Ethics Committee of Tokyo Medical University Hospital (approval number: T2020-0437) and adhered to the Declaration of Helsinki (2013). The requirement to obtain informed consent was waived by the Ethics Committee of Tokyo Medical University Hospital due to the retrospective study design.

### Study participants

COVID-19 patients who were treated in the ICU with IMV were included in this study. Patients with missing body mass index (BMI) data were excluded. We used only the data at the first time of ICU admission if patients were admitted to the ICU multiple times. To clarify the association between obesity and outcomes in an Asian population, we also excluded the non-Asian population in this study.

### Data collection and definitions

Patient’s age, sex, height, body weight, racial category, coexisting disorder (chronic pulmonary disease, chronic kidney disease, congestive heart failure, and diabetes), Charlson Comorbidity Index (CCI)^15^, vital signs at presentation, sequential organ failure assessment (SOFA) score^16^, laboratory data on the day of admission, data on arterial blood gases (ABG) (immediately before IMV, after initiating IMV, and immediately before venovenous extracorporeal membrane oxygenation [VV-ECMO]), data on respiratory therapy before IMV, treatment details during the hospital stay, partial pressure of arterial oxygen/fraction of inspiratory oxygen ratio just before intubation, parameters of ventilator (at initiation of IMV and immediately before VV-ECMO), number of ventilator-free days [VFDs] at 28 days^17^, length of ICU stay, in-hospital mortality, and requirement for VV-ECMO treatment were retrieved from the J-RECOVER study^13^. BMI was calculated by using the following formula: BMI = kg/m^2^. Based on previous reports of low BMI in the Japanese population^18^, obesity was defined as BMI ≥ 25 kg/m^2^ according to the definition of the Japan Society for the Study of Obesity^19^. BMI values that appeared to be abnormal were reconfirmed with each facility.

### Outcomes

The primary outcome was in-hospital mortality. The secondary outcome was the requirement of VV-ECMO treatment.

### Statistical analysis

Univariate and multivariate logistic regression models were created to determine the association between obesity and the outcome. Age, sex, and CCI were used in the multivariate model to adjust the baseline characteristics based on previous studies^8,11,20^. Complete case analysis was performed. We performed a sensitivity analysis by adding the SOFA score as a covariate to adjust the baseline severity according to the previous reports^11,12^. Additionally, sensitivity analysis was performed by adding diabetes as a covariate based on previously reported results^21^. Furthermore, given the national differences in obesity definition, a sensitivity analysis was conducted using the Center for Disease Control and Prevention (CDC) definition of obesity, i.e., BMI ≥ 30 kg/m^2^, as the cutoff^22^. According to the result of the multivariate analysis, we created a scatter plot to show the factors that might have affected the association between in-hospital mortality and BMI. To visualize the association between obesity and mortality, spline curves were generated using a generalized additive model (GAM) with unadjusted and adjusted models (adjusted for age, sex, and CCI).

The Mann–Whitney *U* or Fisher’s exact test was used for the comparison between the obesity and nonobesity groups. Data were described using median and interquartile range (IQR) for continuous variables and exact number and percentage (%) for categorical variables. A two-sided p value of < 0.05 was considered significant. All analyses were performed with R statistical software version 4.0.3 (R Foundation for Statistical Computing).

## Results

**Figure 1:**
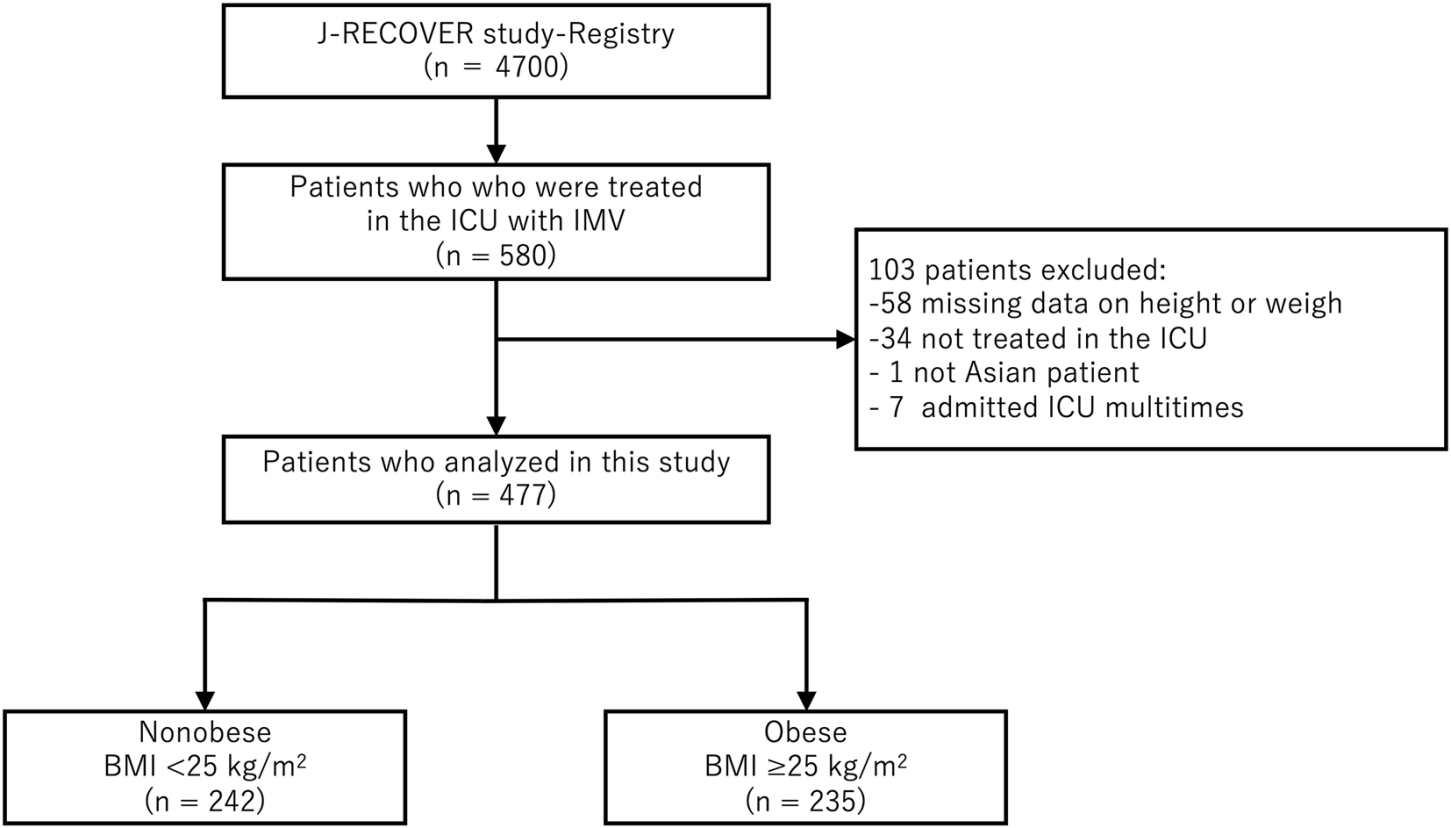
Flow chart of inclusion in the present study. *J-RECOVER study* Japanese multicenter research of COVID-19 by assembling real-world data registry, *ICU* intensive care unit, *IMV* invasive mechanical ventilation, *BMI* body mass index.

Figure 1 shows the flowchart of this study. The obese (BMI ≥ 25 kg/m^2^) and nonobese (BMI < 25 kg/m^2^) groups included 235 and 242 patients, respectively. There were 77 and 17 patients with BMI ≥ 30 kg/m^2^ and ≥ 35 kg/m^2^, respectively. A histogram presenting the distribution of BMI is shown in Supplementary Fig. S1.

Table 1 details the characteristics of the participants. Treatment details are shown in Supplementary Table S1. Overall, the study participants were predominantly male individuals (78.4%). No patient had a history of chronic lung disease. The median BMI (IQR) in the whole population was 25.0 (22.3–28.1) kg/m^2^. The median BMI (IQR) of the obese and nonobese groups was 28.2 (26.6–31.2) and 22.4 (20.7–23.7) kg/m^2^, respectively. The obese group was significantly younger (61 [IQR 51–71] vs. 70 [IQR 63–77] years, p < 0.010). There were no significant differences between the two groups in CCI (0 [IQR 0–1] vs. 0 [IQR 0–1], p = 0.689) and SOFA scores (4 [IQR 2–7] vs. 4 [IQR 3–7], p = 0.184). The data of ABG and ventilator settings are shown in Supplementary Table S2a,b. There was no significant difference between the two groups in the number of VFDs at 28 days (7 [IQR 0–19] vs. 9 [IQR 0–21] days, p = 0.655) and length of ICU stay (13 [IQR 8–22] vs. 13 [IQR 8–21] days, p = 0.401). Table 2 details the outcome of the participants. The obese group had a lower proportion of in-hospital deaths (20.9% vs. 29.3%, p = 0.035) and a greater implementation of VV-ECMO than the nonobese group (22.1% vs. 15.7%, p = 0.080).

**Table 1:** Characteristics of patients with COVID-19 who required mechanical ventilation stratified by BMI.

Variables	Overall		Nonobese (BMI < 25 kg/m^2^)	Obese (BMI ≥ 25 kg/m^2^)	p value
	n = 477	Missing (%)	n = 242	n = 235	
Age (year)	67 (56–75)	–	70 (63–77)	61 (51–71)	< 0.001
Male sex (%)	374 (78.4)	–	186 (77.9)	188 (80.0)	0.437
BMI (kg/m^2^)	25.0 (22.3–28.1)	–	22.4 (20.7–23.7)	28.2 (26.6–31.2)	< 0.001
Coexisting disorder (%)					
Chronic pulmonary disease	0 (0)	–	0 (100)	0 (100)	NA
Chronic kidney disease	19 (4.00)	–	14 (5.80)	5 (2.10)	0.059
Congestive heart failure	15 (3.10)	–	11 (4.50)	4 (1.70)	0.113
Diabetes	133 (27.9)	–	55 (22.7)	78 (33.2)	0.014
CCI	0 (0, 1)	–	0 (0, 1)	0 (0, 1)	0.689
Vital signs on admission					
SBP (mmHg)	130.0 (114.0–152.0)	0.63	128.0 (112.8–151.3)	131.0 (117.0–151.0)	0.410
DBP (mmHg)	75.0 (64.0–85.0)	0.84	73.0 (62.0–83.0)	78.0 (64.8–88.0)	0.027
Pulse rate (bpm)	90 (76–103)	1.1	86 (75–103)	91 (78–103)	0.136
Respiratory rate (bpm)	22 (18–26)	10.9	22 (18–26)	22 (18–27)	0.346
Glasgow coma scale	15.0 (8.0–15.0)	2.1	15.0 (7.5–15.0)	15.0 (9.75–15.0)	0.037
Body temperature (°C)	37.2 (36.6–38.1)	0.42	37.1 (36.5–38.0)	37.5 (36.6–38.3)	0.008
SOFA score on admission	4 (3–7)	21.4	4 (3–7)	4 (2–7)	0.184
LD at the time of admission					
WBC count (× 10^3^/μL)	7.1 (5.4–10.1)	17.2	7.3 (5.5–10.7)	7.0 (5.1–9.5)	0.333
Platelet count (× 10^4/^μL)	19.0 (14.8–25.7)	17.2	18.3 (14.6–25.6)	19.5 (15.1–25.6)	0.401
Creatinine (mg/dL)	0.87 (0.70–1.15)	17.2	0.84 (0.69–1.14)	0.90 (0.73–1.15)	0.235
Total bilirubin (mg/dL)	0.60 (0.40–0.80)	18.9	0.60 (0.40–0.80)	0.60 (0.40–0.80)	0.633
CRP (mg/dL)	11.5 (6.1–17.1)	20.3	12.56 (6.84–18.52)	10.06 (5.45–15.74)	0.007
d-Dimer (µg/mL)	1.7 (1.0–4.8)	24.9	2.2 (1.2–7.2)	1.4 (0.9–3.3)	< 0.001
Lactic acid (mmol/L)	1.6 (1.1–6.0)	25.6	1.5 (1.1–7.0)	1.7 (1.2–5.0)	0.391
Respiratory therapy before IMV initiation (%)					
HFNC	20 (4.19)	–	8 (3.31)	12 (5.11)	0.367
NPPV	3 (0.63)	–	1 (0.41)	2 (0.85)	0.619
P/F ratio at time of IMV initiation	130.5 (103.5–170.7)	52.8	130.1 (95.8–168.3)	130.5 (106.2–171.7)	0.687

Data are presented as median (IQR) for continuous variables.

*BMI* body mass index, *CCI* Charlson Comorbidity Index, *SBP* systolic blood pressure, *DBP* diastolic blood pressure, *SOFA* *score* sequential organ failure assessment score, *LD* laboratory data, *IMV* invasive mechanical ventilation, *WBC* white blood cell count, *CRP* C-reactive protein, *IMV* invasive mechanical ventilation, *HFNC* high-flow nasal cannula, *NPPV* noninvasive positive pressure ventilation, *P/F* PaO_2_/FiO_2_.

**Table 2:** Outcome of patients with COVID-19 who required mechanical ventilation stratified by BMI.

Outcome variables	Overall	Nonobese (BMI < 25 kg/m^2^)	Obese (BMI ≥ 25 kg/m^2^)	p value
	n = 477	n = 242	n = 235	
In-hospital death (%)	120 (25.2)	71 (29.3)	49 (20.9)	0.035
VV-ECMO (%)	90 (18.9)	38 (15.7)	52 (22.1)	0.080

*BMI* body mass index, *VV-ECMO* venovenous extracorporeal membrane oxygenation.

**Figure 2:**
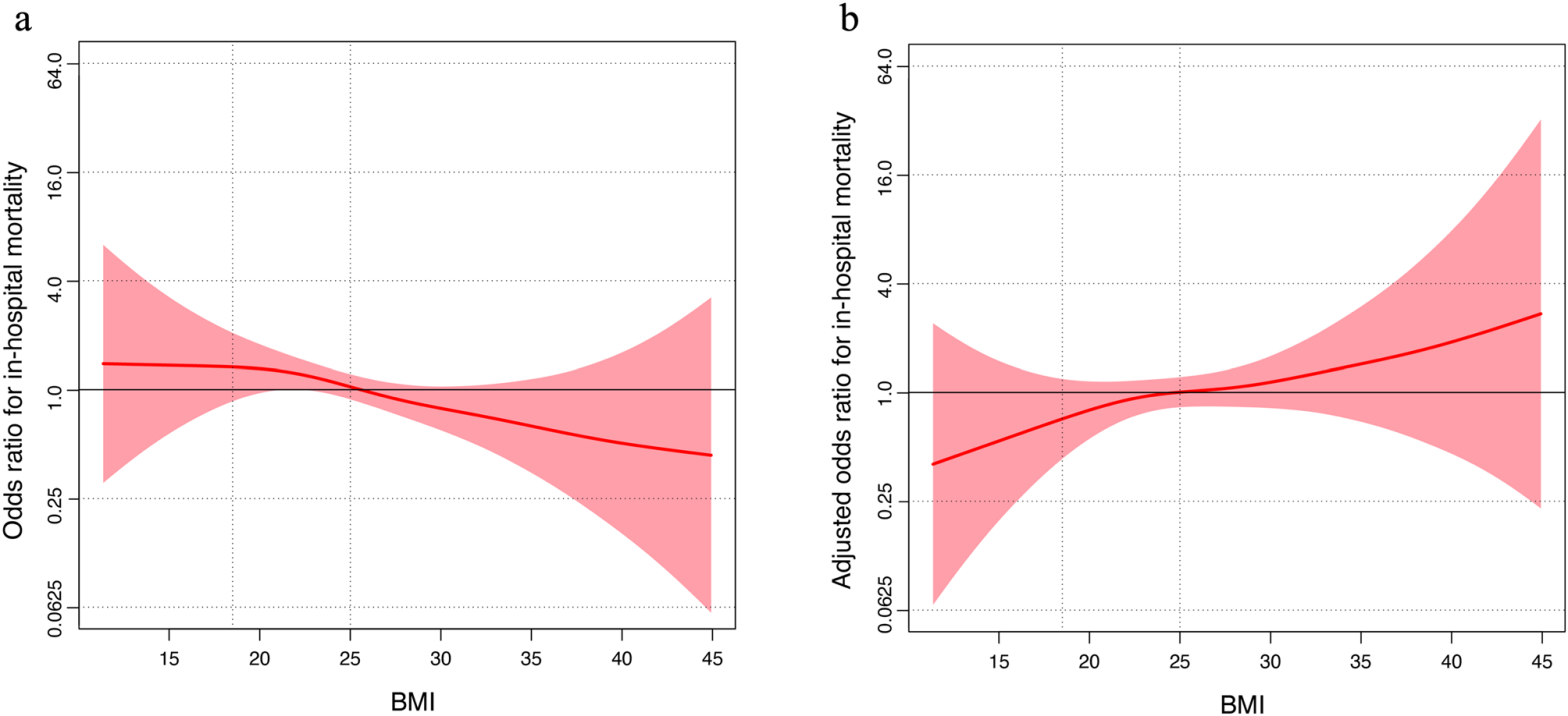
The spline curve generated using GAMs. (**a**) The spline curve of the univariate model along with standard error. (**b**) The spline curve of the multivariate model with age, male sex, and CCI as covariates along with the standard error. *GAMs* generalized additive models, *CCI* Charlson Comorbidity Index.

The spline curve generated using GAM is shown in Fig. 2a,b. In the univariate model, the curve showed that the odds ratio (OR) for mortality decreased with increasing BMI (Fig. 2a), while the adjusted OR for death increased with increasing BMI in the multivariate model (Fig. 2b).

The univariate logistic regression model showed that obesity was statistically significantly associated with lower in-hospital mortality (OR 0.63, 95% confidence interval [CI 0.42–0.97], p = 0.033). Contrarily, the multivariate logistic regression model adjusted for age, sex, and CCI as covariates showed no significant association between obesity and in-hospital mortality (OR 1.15, [95% CI 0.72–1.84], p = 0.564) (Table 3a). Since the multivariate logistic regression model showed the disappearance of a significant association between obesity and mortality but showed a significant association between age and mortality, we described the scatter plot for BMI and age (Supplementary Fig. S2); this plot showed that the BMI decreases with increasing patient age.

**Table 3:** Univariate and multivariate associations of obesity (BMI ≥ 25 kg/m^2^) with (a) in-hospital mortality and (b) with VV-ECMO.

Variables	Univariate	p value	Multivariate	p value
	OR (95% CI)		OR (95% CI)	
(a) In-hospital mortality				
Obese	0.634 (0.417–0.965)	0.033	1.150 (0.717–1.840)	0.564
Age	–		1.090 (1.070–1.120)	< 0.001
Male sex	–		1.180 (0.685–2.040)	0.546
CCI	–		1.140 (0.908–1.440)	0.253
(b) With VV-ECMO				
Obese	1.530 (0.960–2.420)	0.074	1.110 (0.669–1.830)	0.695
Age	–		0.962 (0.945–0.980)	< 0.001
Male sex	–		1.030 (0.564–1.890)	0.920
CCI	–		0.705 (0.516–0.963)	0.027

Multivariate regression model was adjusted with age, male sex, and CCI.

*BMI* body mass index, *OR* odds ratio, *CI* confidence interval, *CCI* Charlson Comorbidity Index, *VV-ECMO* venovenous extracorporeal membrane oxygenation.

There were no significant associations between obesity and the implementation of VV-ECMO in both the univariate and multivariate models (univariate OR 1.53, [95% CI 0.96–2.42], p = 0.074; multivariate OR 1.11, [95% CI 0.67–1.83], p = 0.695) (Table 3b).

The sensitivity analysis was adjusted for factors, including the SOFA score or diabetes, and similar results were obtained for both in-hospital mortality and VV-ECMO implementation (Supplementary Table S3). Supplementary Table S4 presents the characteristics of the participants at the BMI cutoff ≥ 30 kg/m^2^. The analysis using this cutoff revealed difference in CCI. The sensitivity analysis using a BMI cutoff of ≥ 30 kg/m^2^ for obesity indicated that obesity did not have a significant association with in-hospital mortality or VV-ECMO implementation (Supplementary Table S5).

## Discussion

In this study, we examined the associations between obesity and in-hospital mortality and VV-ECMO implementation in Asian COVID-19 patients requiring IMV. Univariate analysis showed that obesity was significantly associated with lower in-hospital mortality; however, this association disappeared in the multivariate analysis. In addition, obesity was not associated with the implementation of VV-ECMO.

In COVID-19, obesity has been reported as a risk factor for increased severity^5,6,8,11,12^. This has been explained by several mechanisms, including the causal relationship between obesity and multiple comorbidities such as hypertension, diabetes, and thrombosis^23^, decreased functional residual capacity, and increased respiratory workload^24^. In addition, the fact that fat enhances AT2 expression^25^ and COVID-19 also enhances AT2 expression by down-regulation of ACE2, which may synergistically promote pathologic injuries in the lungs by exerting proinflammatory responses and increasing vascular permeability^26–28^. There are limited studies investigating the association between obesity and prognosis, including mortality, in severe COVID-19, and it is unclear whether there is an association between them. A large observational study^29^ of 2635 COVID-19 patients who were admitted to the ICU reported that obesity did not affect ICU mortality, which was similar to the findings of the present study. Some previous multicenter studies^11,30,31^ of COVID-19 patients admitted to the ICU reported different results, with obesity significantly associated with increased mortality. However, the cutoff values for obesity shown to be associated with death were BMI of 30, 35, and 40 kg/m^2^, and no association with death was found below these relatively large BMI, which is similar to the results of this study. Contrarily, Richard et al.^12^ reported different results from those of the present study in their observational study of 222 severe COVID-19 patients, showing an obesity paradox with the lowest OR for death in BMI between 30 and 40 kg/m^2^ in a multivariate analysis. However, this study included 4.5% of highly probable COVID-19. Although these studies^11,12,29–31^ were conducted in severe COVID-19 patients admitted to the ICU, they also included non-IMV patients. Since the indication for ICU admission is largely affected by the supply and demand balance of medical care, especially in a pandemic situation, it would be inappropriate to define patient severity by ICU admission. In the present study, we analyzed only critically ill patients by selecting patients who required IMV. Therefore, the result of the present study would extend the evidence for predicting outcomes in severely ill COVID-19 patients, along with previous reports.

In this study, the association between obesity and low in-hospital mortality disappeared when covariates were considered. First, there were few patients with very high BMI in the Asian population represented by this study, as compared to previously reported cases^12,29,30^. As shown in the Supplementary Figs. 1 and 2, similar to previous reports of low obesity rates in the Japanese population^18^, there are not many obese patients with BMI > 30 kg/m^2^ evaluated in this study, which may have influenced the present results. The fact that the standard error generated using GAM becomes larger with increasing BMI also indicates that there are few patients with large BMI. Furthermore, this factor could also be attributed to the lack of association between obesity and the adoption rate of VV-ECMO. Previous study suggested that severe obesity (BMI > 40 kg/m^2^) was a relative contraindication^32^, which could have impacted the implementation of VV-ECMO in obese patients. However, in this cohort, the prevalence of severe obesity was remarkably low, suggesting its limited influence. The second mechanism is thought to be the younger age of the obese group and a large number of elderly patients in the nonobese group. Gupta et al.^10^ reported that age was the most important factor affecting mortality in patients with COVID-19 requiring ICU admission. This is consistent with a lower OR for death due to the younger age of the obesity group. Furthermore, the obese group did not show any difference in the prevalence of coexisting disorders, which may have influenced the results. In the Asian populations, the distribution of visceral adipose tissue (VAT) is greater than that of subcutaneous fat with the same BMI, and they are more likely to develop lifestyle-related diseases as compared to western populations^33–35^. Additionally, this cohort predominantly comprised male participants, who are more likely to have increased VAT than females^36^; however, gender differences did not influence the results in the multivariate analysis. Although the results differ from the present study, it is considered that the obese group was still young and hence less affected by VAT, such as lifestyle-related diseases. Additionally, obese patients may have started intensive care earlier based on the prediction that their illness may become severe, which could have influenced the results. Therefore, in the populations included in this study, in COVID-19 patients requiring IMV, obesity does not appear to have a strong effect on mortality. Clinicians treating these patients may be aware that BMI is not a strong predictor of mortality.

### Strengths and limitations

Our study has several notable strengths. First, to the best of our knowledge, this is the first study that investigated the association between obesity and mortality using real-world data from a large cohort of confirmed COVID-19 patients requiring IMV in an Asian population. The current study findings will contribute to further research in this population. Second, this study used a unified format to obtain information on the course of hospitalization at 66 research and teaching hospitals across Japan; thus, a certain level of generalizability was expected.

However, there are several limitations of this study. First, due to a real-world study with a retrospective design, there is a concern regarding the inaccuracy of follow-up evaluation compared to a prospective design. Moreover, treatments were not standardized. Thus, the issue of residual confounding would be a major limitation. Therefore, future studies with prospective design are warranted. Second, some of the BMI data included self-reported values, and the methods of measuring height and weight are not standardized in each hospital, which may introduce measurement bias. Furthermore, this study was based on relatively early data in the COVID-19 epidemic. Considering the emergence of different virus variants and wide availability of vaccines, the results of this study must be cautiously interpreted in future epidemics. Moreover, continuous collection of data in this regard is also warranted.

In conclusion, this Japanese multicenter study on patients with severe COVID-19 requiring IMV showed that obesity was not associated with in-hospital mortality. Obesity may not be regarded as a failure factor in the intensive care of critically ill COVID-19 patients.

## Supplementary Information

Supplementary Figures.

Supplementary Tables.

## Data Availability

The datasets used and/or analyzed during the current study are available from the corresponding author upon reasonable request.

## References

[1] World Health Organization. WHO coronavirus (COVID-19) dashboard in *WHO Health Emergency Dashboard* (World Health Organization, 2022). https://covid19.who.int/.

[2] COVID-19 Excess Mortality Collaborators (2022). Estimating excess mortality due to the COVID-19 pandemic: A systematic analysis of COVID-19-related mortality, 2020–21. Lancet.

[3] Richardson S (2020). Presenting characteristics, comorbidities, and outcomes among 5700 patients hospitalized with COVID-19 in the New York City area. JAMA.

[4] Lim ZJ (2021). Case fatality rates for patients with COVID-19 requiring invasive mechanical ventilation. A meta-analysis. Am. J. Respir. Crit. Care Med..

[5] Abumayyaleh M (2021). Does there exist an obesity paradox in COVID-19? Insights of the international HOPE-COVID-19-registry. Obes. Res. Clin. Pract..

[6] Biscarini S (2020). The obesity paradox: Analysis from the SMAtteo Covid-19 REgistry (SMACORE) cohort. Nutr. Metab. Cardiovasc. Dis..

[7] Gao M (2021). Associations between body-mass index and COVID-19 severity in 6·9 million people in England: A prospective, community-based, cohort study. Lancet Diabetes Endocrinol..

[8] Palaiodimos L (2020). Severe obesity, increasing age and male sex are independently associated with worse in-hospital outcomes, and higher in-hospital mortality, in a cohort of patients with COVID-19 in the Bronx, New York. Metabolism.

[9] Williamson EJ (2020). Factors associated with COVID-19-related death using OpenSAFELY. Nature.

[10] Gupta S (2020). Factors associated with death in critically ill patients with coronavirus disease 2019 in the US. JAMA Intern. Med..

[11] Chetboun M (2021). BMI and pneumonia outcomes in critically ill COVID-19 patients: An international multicenter study. Obesity (Silver Spring).

[12] Dana R (2021). Obesity and mortality in critically ill COVID-19 patients with respiratory failure. Int. J. Obes. (Lond.).

[13] Tagami T (2022). Japanese multicenter research of COVID-19 by assembling real-world data: A study protocol. ACE.

[14] Yasunaga H (2019). Real world data in Japan: Chapter II the diagnosis procedure combination database. ACE.

[15] Charlson ME, Pompei P, Ales KL, MacKenzie CR (1987). A new method of classifying prognostic comorbidity in longitudinal studies: Development and validation. J. Chronic Dis..

[16] Vincent JL (1996). The SOFA (sepsis-related organ failure assessment) score to describe organ dysfunction/failure. On behalf of the Working Group on Sepsis-Related Problems of the European Society of Intensive Care Medicine. Intensive Care Med..

[17] Schoenfeld DA, Bernard GR, ARDS Network (2002). Statistical evaluation of ventilator-free days as an efficacy measure in clinical trials of treatments for acute respiratory distress syndrome. Crit. Care. Med..

[18] World Health Organization. Global Health Observatory data repository, Prevalence of obesity among adults, BMI ≥ 30, age-standardized Estimates by country. https://apps.who.int/gho/data/node.main.A900A?lang=en/.

[19] Japan Obesity Society. Obesity clinical practice guidelines 2022. Guidelines for the management of obesity disease, 2022. http://www.jasso.or.jp/contents/magazine/journal.html.

[20] Nakeshbandi M (2020). The impact of obesity on COVID-19 complications: A retrospective cohort study. Int. J. Obes. (Lond.).

[21] Dennis JM (2021). Type 2 diabetes and COVID-19-related mortality in the critical care setting: A National Cohort Study in England, March–July 2020. Diabetes Care.

[22] Center for Disease Control and Prevention. Defining Adult Overweight and Obesity. https://www.cdc.gov/obesity/basics/adult-defining.html.

[23] Sattar N, McInnes IB, McMurray JJV (2020). Obesity is a risk factor for severe COVID-19 infection: Multiple potential mechanisms. Circulation.

[24] Pépin JL (2016). Prevention and care of respiratory failure in obese patients. Lancet Respir. Med..

[25] Sharma AM (2004). Is there a rationale for angiotensin blockade in the management of obesity hypertension?. Hypertension.

[26] Tartof SY (2020). Obesity and mortality among patients diagnosed with COVID-19: Results from an integrated health care organization. Ann. Intern. Med..

[27] Poly TN (2021). Obesity and mortality among patients diagnosed with COVID-19: A systematic review and meta-analysis. Front. Med. (Lausanne).

[28] Bornstein SR, Dalan R, Hopkins D, Mingrone G, Boehm BO (2020). Endocrine and metabolic link to coronavirus infection. Nat. Rev. Endocrinol..

[29] Kooistra EJ (2022). Body mass index and mortality in coronavirus disease 2019 and other diseases: A cohort study in 35,506 ICU patients. Crit. Care Med..

[30] COVID-ICU Group on behalf of the REVA Network and the COVID-ICU Investigators (2021). Clinical characteristics and day-90 outcomes of 4244 critically ill adults with COVID-19: A prospective cohort study. Intensive Care Med..

[31] Sjögren L (2021). Impact of obesity on intensive care outcomes in patients with COVID-19 in Sweden—a cohort study. PLoS One.

[32] Shekar K (2020). Extracorporeal life support organization coronavirus disease 2019 interim guidelines: A consensus document from an International Group of Interdisciplinary Extracorporeal Membrane Oxygenation Providers. ASAIO J..

[33] Fujimoto WY (2000). Preventing diabetes—applying pathophysiological and epidemiological evidence. Br. J. Nutr..

[34] Oka R (2010). Impacts of visceral adipose tissue and subcutaneous adipose tissue on metabolic risk factors in middle-aged Japanese. Obesity (Silver Spring).

[35] Tang L, Zhang F, Tong N (2016). The association of visceral adipose tissue and subcutaneous adipose tissue with metabolic risk factors in a large population of Chinese adults. Clin. Endocrinol. (Oxf.).

[36] Demerath EW (2007). Anatomical patterning of visceral adipose tissue: Race, sex, and age variation. Obesity.

