# Supplementary material for: Association between obesity and mortality in critically ill COVID-19 patients requiring invasive mechanical ventilation: a multicenter retrospective observational study
Source: Sci Rep. 2023 Jul 24;13:11961. doi: 10.1038/s41598-023-39157-8 (PMC10366113; doi:10.1038/s41598-023-39157-8)
Supplement: Supplementary file 1 — Supplementary Figures. [file 41598_2023_39157_MOESM1_ESM.pdf]

Title: Association between obesity and mortality in critically ill COVID-19 patients requiring invasive mechanical ventilation: A multicenter retrospective observational study

Authors:

Keiichiro Shimoyama, MD; Akira Endo, MD, PhD; Takashi Shimazui, MD, PhD; Takashi Tagami, MD, MPH, PhD; Kazuma Yamakawa, MD, PhD; Mineji Hayakawa, MD, PhD; Takayuki Ogura, MD, PhD; Atsushi Hirayama, MD, PhD; Hideo Yasunaga, MD, PhD; Jun Oda, MD, PhD

Supplemental Fig. 1 Histogram of BMI

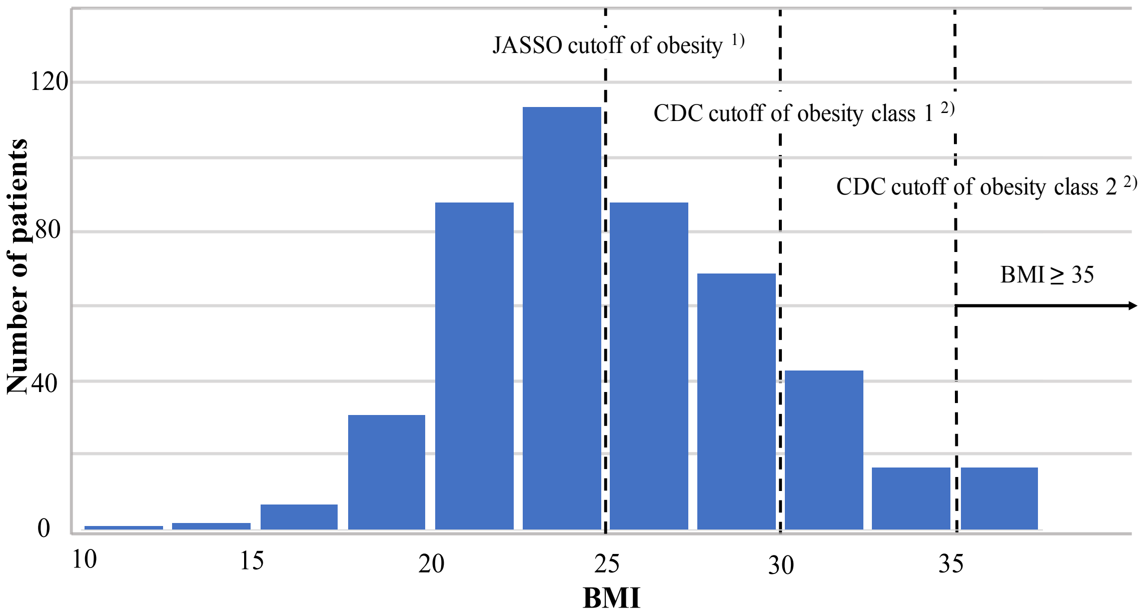

Abbreviations: BMI, body mass index; JASSO, japan society for the study of obesity; CDC, center for disease control and prevention

Supplemental Fig. 2 Scatter plots for age and BMI

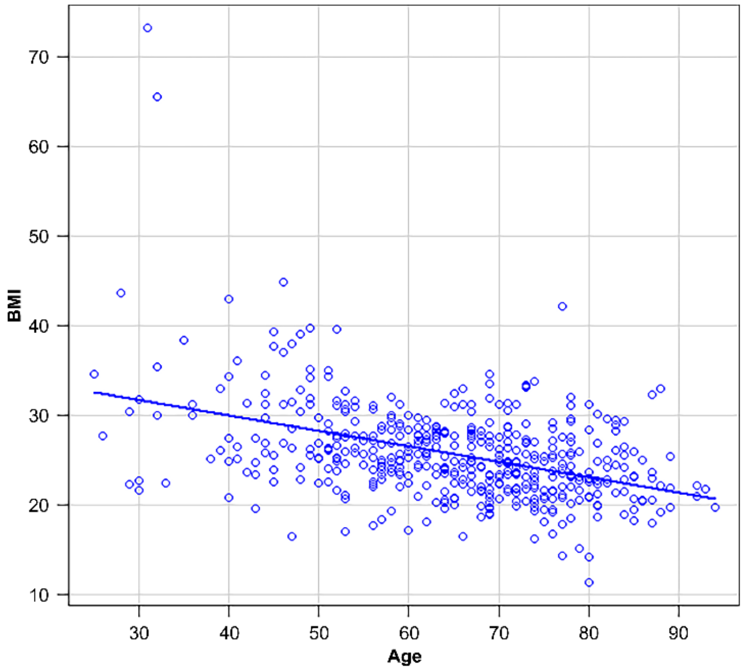

Abbreviations: BMI, body mass index

References

1. Japan Obesity Society. Obesity clinical practice guidelines 2022. Guidelines for the management of obesity disease, 2022, Available from: http://www.jasso.or.jp/contents/magazine/journal.html

2. Center for Disease Control and Prevention. Defining Adult Overweight & Obesity, Available from: https://www.cdc.gov/obesity/basics/adult-defining.html
