# Supplementary material for: Association between obesity and mortality in critically ill COVID-19 patients requiring invasive mechanical ventilation: a multicenter retrospective observational study
Source: Sci Rep. 2023 Jul 24;13:11961. doi: 10.1038/s41598-023-39157-8 (PMC10366113; doi:10.1038/s41598-023-39157-8)
Supplement: Supplementary file 2 — Supplementary Tables. [file 41598_2023_39157_MOESM2_ESM.pdf]

Title: Association between obesity and mortality in critically ill COVID-19 patients requiring invasive mechanical ventilation: A multicenter retrospective observational study

Authors:

Keiichiro Shimoyama, MD; Akira Endo, MD, PhD; Takashi Shimazui, MD, PhD; Takashi Tagami, MD, MPH, PhD; Kazuma Yamakawa, MD, PhD; Mineji Hayakawa, MD, PhD; Takayuki Ogura, MD, PhD; Atsushi Hirayama, MD, PhD; Hideo Yasunaga, MD, PhD; Jun Oda, MD, PhD

Supplementary Table S1. Treatment and clinical course of patients with COVID-19 who required mechanical ventilation stratified on the basis of body mass index

	**Overall**	**Nonobese (BMI < 25 kg/m²)**	**Obese (BMI ≥ 25 kg/m²)**	**p-value**
**Variables**	n = 477	n = 242	n = 235	
Steroid (%)	217 (45.5)	115 (47.5)	102 (43.4)	0.408
Anticoagulation (%)	341 (71.5)	171 (70.7)	170 (72.3)	0.761
Remdesivir (%)	111 (23.3)	53 (21.9)	58 (24.7)	0.516
Sedation (%)	267 (56.0)	137 (56.6)	130 (55.3)	0.783
Neuromuscular-blocking (%)	184 (38.6)	99 (40.9)	85 (36.2)	0.302
Prone positioning (%)	180 (37.7)	93 (41.9)	87 (39.4)	0.629
Nitric oxide inhalation (%)	16 (3.4)	10 (4.1)	6 (2.6)	0.447
Tracheostomy (%)	106 (22.2)	61 (25.2)	45 (19.1)	0.124

Abbreviations: BMI, body mass index; ICU, intensive care unit

Supplementary Table S2

a. Arterial blood gas analysis data and initial ventilator settings in patients with COVID-19 who required mechanical ventilation stratified on the basis of body mass index, including percentages of missing values

	Overall		Nonobese (BMI < 25 kg/m^2)	Obese (BMI ≥ 25 kg/m^2)	p-value
**Variables**	n = 477	Missing (%)	n = 242	n = 235	
ABG just before starting IMV					
pH	7.44 (7.40–7.48)	52.0	7.44 (7.39–7.48)	7.44 (7.41–7.47)	0.906
PaO_2 (mmHg)	69.7 (58.0–86.7)	51.6	97.1 (76.4–139.0)	91.3 (75.1–118.1)	0.048
$PaCO_2$ (mmHg)	34.4 (30.8–38.0)	51.4	43.2 (38.0–50.0	42.2 (37.3–48.5)	0.323
Base excess	−1.0 (−2.9–1.7)	52.2	−0.10 (−2.50–1.70)	−0.1 (−2.9–1.7)	0.923
Initial ABG after starting IMV					
pH	7.36 (7.30–7.41)	6.7	7.35 (7.30–7.41)	7.37 (7.31–7.41)	0.298
PaO_2 (mmHg)	93.1 (75.6–128.0)	6.7	68.7 (57.4–86.2)	71.5 (60.0–86.5)	0.443
$PaCO_2$ (mmHg)	42.7 (37.4–49.8)	6.7	33.9 (30.8–38.0)	35.1 (31.3–38.0)	0.445
Base excess	−1.4 (−3.8–0.9)	7.1	−1.6 (−4.0–0.8)	−1.3 (−3.7–0.9)	0.328
First settings of IMV					0.410
FiO_2	0.60 (0.50–0.80)	5.7	0.60 (0.50–0.80)	0.60 (0.50–0.90)	0.081
Peak inspiratory pressure (cmH_2O)	24.0 (20.0–26.0)	14.9	22.5 (20.0–26.0)	24.0 (21.0–27.0)	0.014
Mean airway pressure	14.0 (12.0–16.7)	43.8	14.0 (12.0–16.0)	15.0 (12.0–17.0)	0.067
Tidal volume (ml)	429.5 (380.8–500.0)	20.8	420.0 (380.0–480.0)	450.0 (396.3–505.0)	0.029
Respiratory rate (bpm)	16 (14–20)	14.9	18 (15–20)	16 (14–20)	0.353

Abbreviations: BMI, body mass index; ABG, arterial blood gas analysis; IMV, invasive mechanical ventilation; PaO_2, partial pressure of arterial oxygen; $PaCO_2$, partial pressure of arterial carbon dioxide

b. Arterial blood gas analysis data and ventilator settings immediately before VV-ECMO initiation in patients with COVID-19 who required mechanical ventilation stratified on the basis of body mass index, including percentages of missing values

Variables	Overall n = 90	Missing (%)	Nonobese (BMI < 25 kg/m^2) n = 38	Obese (BMI ≥ 25 kg/m^2) n = 52	p-value
ABG just before starting VV-ECMO					
pH	7.35 (7.24–7.42)	7.8	7.34 (7.26–7.41)	7.35 (7.24–7.42)	0.941
PaO_2 (mmHg)	71.3 (62–87.1)	7.8	69.1 (57.8–76.6)	75.8 (62.5–95.5)	0.038
$PaCO_2$ (mmHg)	45.4 (40.4–59.3)	7.8	45.8 (40.9–60.7)	45.4 (39.6–58.5)	0.636
Base excess	−0.3 (−3.0–2.6)	7.8	−0.3 (−2.6–2.6)	−0.3 (−3.1–2.6)	0.789
Settings of IMV just before starting VV-ECMO					
FiO_2	0.85 (0.70–1.00)	6.7	0.80 (0.60–1.00)	0.97 (0.74–1.00)	0.273
Peak inspiratory pressure (cmH_2O)	26 (22–29.3)	17.8	25.0 (22.0–28.0)	27.0 (23.8–30.0)	0.194
Mean airway pressure	18 (15.5–20.5)	54.4	19.0 (16.3–20.4)	18.0 (15.5–20.2)	0.947
Tidal volume (ml)	422.5 (380.0–500.0)	15.6	430.0 (380.8–504.8)	411.0 (379.3–490.0)	0.535
Respiratory rate (bpm)	19 (15–24)	16.7	20.0 (15.0–24.0)	18.5 (15.3–24.0)	0.932
PEEP	12 (10–15)	8.9	12.0 (10.0–15.0)	12.0 (10.0–15.0)	0.403

Abbreviations: BMI, body mass index; VV-ECMO, venovenous extracorporeal membrane oxygenation; ABG, arterial blood gas analysis; PaO_2, partial pressure of arterial oxygen; $PaCO_2$, partial pressure of arterial carbon dioxide; IMV, invasive mechanical ventilation; PEEP, positive end-expiratory pressure

Supplementary Table S3

a. Multivariate sensitivity analysis to determine the association of obesity (BMI ≥ 25 kg/m^2) with in-hospital mortality

	Model 1		Model 2	
	Multivariate		Multivariate	
**Variables**	**OR** (95%CI)	**p-value**	**OR** (95%CI)	**p-value**
Obesity	1.630 (0.940–2.840)	0.082	1.210 (0.751–1.960)	0.429
Age	1.100 (1.070–1.130)	<0.001	1.090 (1.070–1.120)	<0.001
Male sex	0.966 (0.520–1.790)	0.912	1.180 (0.685–2.050)	0.545
CCI	1.180 (0.899–1.550)	0.233	1.320 (0.985–1.770)	0.064
SOFA	1.130 (1.040–1.230)	0.005	-	
Diabetes	-		0.611 (0.316–1.190)	0.145

Abbreviations: BMI, body mass index; OR, odds ratio; CI, confidence interval; CCI, Charlson Comorbidity Index; SOFA, sequential organ failure assessment

Model 1 was adjusted with age, male sex, CCI, and SOFA score as covariates.

Model 2 was adjusted with age, male sex, CCI, and diabetes as covariates.

b. Multivariate sensitivity analysis to determine the association of obesity (BMI ≥ 25 kg/m^2) with VV-ECMO

	Model 1		Model 2	
	Multivariate		Multivariate	
**Variables**	**OR** (95%CI)	**p-value**	**OR** (95%CI)	**p-value**
Obesity	1.050 (0.570–1.920)	0.884	1.110 (0.672–1.850)	0.676
Age	0.959 (0.937–0.981)	<0.001	0.962 (0.945–0.980)	<0.001
Male sex	0.930 (0.457–1.900)	0.843	1.030 (0.563–1.890)	0.923
CCI	0.764 (0.537–1.090)	0.134	0.731 (0.474–1.130)	0.154
SOFA	1.320 (1.210–1.450)	<0.001	-	

Diabetes	-	0.910 (0.405–2.040)	0.819

Abbreviations: BMI, body mass index; VV-ECMO, venovenous extracorporeal membrane oxygenation; OR, odds ratio; CI, confidence interval; CCI, Charlson Comorbidity Index; SOFA, sequential organ failure assessment

Model 1 was adjusted with age, male sex, CCI, and SOFA score as covariates.

Model 2 was adjusted with age, male sex, CCI, and diabetes as covariates.

Supplementary Table S4. Characteristics and laboratory data of patients with COVID-19 who required mechanical ventilation stratified on the basis of body mass index (cutoff value, 30 kg/m^2)

	**Nonobese (BMI < 30 kg/m^2)**	**Obese (BMI ≥ 30 kg/m^2)**	**p-value**
**Variables**	n = 400	n = 77	
Age (year)	68 (59–76)	52 (45–67)	<0.001
Male sex (%)	318 (79.5)	56 (72.7)	0.225
BMI (kg/m^2)	24.2 (21.8–26.5)	32.0 (31.2–34.6)	<0.001
Coexisting disorder (%)			
Chronic pulmonary disease	0 (100)	0 (100)	NA
Chronic kidney disease	15 (3.8)	4 (5.2)	0.527
Congestive heart failure	13 (3.2)	2 (2.6)	1.000
Diabetes	102 (25.5)	31 (40.3)	0.012
CCI	0 (0–1)	0 (0–1)	0.027
Vital signs on admission			
SBP (mmHg)	130.0 (115.0–52.0)	126 (112.0–152.0)	0.518
DBP (mmHg)	74.0 (63.0–84.0)	78.0 (69.0–90.0)	0.084
Pulse rate (bpm)	89 (76–104)	92 (76–02)	0.718
Respiratory rate (bpm)	22 (18–26)	22 (19–30)	0.242
Glasgow coma scale	15 (8–15)	15.0 (7–15)	0.860
Body temperature (℃)	37.2 (36.5–38.1)	37.5 (36.8–38.4)	0.041
SOFA score on admission	4 (3–7)	4 (3–8)	0.302
LD at the time of admission			
WBC count (×10^3/μL)	7.3 (5.4–10.5)	6.7 (5.2–8.8)	0.228
Platelet count (×10^4/μL)	19.2 (14.9–26.0)	18.5 (13.9–23.2)	0.253
Creatinine (mg/dL)	0.86 (0.69–1.13)	0.90 (0.74–1.24)	0.139
Total bilirubin (mg/dL)	0.60 (0.40–0.80)	0.52 (0.40–0.70)	0.966
CRP (mg/dL)	12.1 (6.6–17.8)	7.2 (4.4–13.9)	0.001

D-dimer (μg/mL)	1.9 (1.1–5.3)	1.2 (0.8–3.1)	0.001
Lactic acid (mmol/L	1.5 (1.1–6.0)	1.6 (1.1–7.5)	0.735
Respiratory therapy before IMV initiation (%)			
HFNC	18 (4.5)	2 (2.6)	0.755
NPPV	2 (0.5)	1 (1.3)	0.411
P/F ratio at time of IMV initiation	126.4 (102.4–169.5)	143.6 (110.4–76.4)	0.262

Abbreviations: BMI, body mass index; CCI, Charlson Comorbidity Index; SBP, systolic blood pressure; DBP, diastolic blood pressure; SOFA score, sequential organ failure assessment score; LD, laboratory data; IMV, invasive mechanical ventilation; WBC, white blood cell count; CRP, C-reactive protein; IMV, invasive mechanical ventilation; HFNC, high-flow nasal cannula; NPPV, noninvasive positive pressure ventilation; P/F, PaO2/FiO2

Data are presented as median (IQR) for continuous variables.

Supplementary Table S5

a. Univariate and multivariate associations of obesity (BMI ≥ 30 kg/m^2) with in-hospital mortality

	Univariate		Multivariate	
**Variables**	**OR** (95% CI)	**p-value**	**OR** (95% CI)	**p-value**
Obesity	0.680 (0.371–1.250)	0.212	1.620 (0.792–3.310)	0.187
Age	-		1.090 (1.070–1.120)	<0.001
Male sex	-		1.230 (0.710–2.130)	0.461
CCI	-		1.130 (0.893–1.420)	0.314

Abbreviations: BMI, body mass index; OR, odds ratio; CI, confidence interval; CCI, Charlson comorbidity index

Multivariate regression model was adjusted with age, male sex, and CCI as covariates.

b. Univariate and multivariate associations of obesity (BMI ≥ 30 kg/m^2) with VV-ECMO

	Model 1 Univariate		Model 2 Multivariate	
**Variables**	**OR** (95%CI)	p-value	**OR** (95%CI)	**p-value**
Obesity	0.947 (0.504–1.780)	0.867	0.529 (0.256–1.090)	0.085
Age	-		0.954 (0.936–0.973)	<0.001
Male sex	-		0.965 (0.524–1.780)	0.921
CCI	-		0.731 (0.536–0.997)	0.048

Abbreviations: BMI, body mass index; VV-ECMO, venovenous extracorporeal membrane oxygenation; OR, odds ratio; CI, confidence interval; CCI, Charlson comorbidity index

Multivariate regression model was adjusted with age, male sex, and CCI as covariates.
